# Efficient Transfer Hydrogenation of Nitro Compounds to Amines Enabled by Mesoporous N-Stabilized Co-Zn/C

**DOI:** 10.3389/fchem.2019.00590

**Published:** 2019-08-27

**Authors:** Yufei Xu, Jingxuan Long, Wenfeng Zhao, Hu Li, Song Yang

**Affiliations:** State Key Laboratory Breeding Base of Green Pesticide & Agricultural Bioengineering, Key Laboratory of Green Pesticide & Agricultural Bioengineering, Ministry of Education, State-Local Joint Laboratory for Comprehensive Utilization of Biomass, Center for Research & Development of Fine Chemicals, Guizhou University, Guiyang, China

**Keywords:** mesoporous bimetallic material, N-doped carbon, transfer hydrogenation, nitrogen-containing compounds, heterogeneous catalyst

## Abstract

N-doped metal materials with enhanced stability and abundant porosity have attracted tremendous attention in catalytic reactions. Herein, a simple solvothermal approach was demonstrated to significantly enlarge the pore dimension of conventional microporous zeolitic imidazolate framework (ZIF) incorporated with two kinds of central metals (Co, Zn), while maintaining the original ZIF crystal morphology. Upon further pyrolysis, the resulting mesoporous Co-Zn/N-C material could possess the highly dispersed metal particle on the N-doped carbon, with satisfactory pore volume and surface area. The partial vaporization of Zn and the stabilizing effect of N, illustrated by XRD, HRTEM, HAADF-STEM with mapping, SEM, Raman Spectrum, BET, and TGA, were able to remarkably increase the accessibility of substrate toward active sites and prevent the aggregation of metal particles, respectively. Under mild reaction conditions, the N-stabilized Co-Zn/N-C exhibited good activity and selectivity in transfer hydrogenation of various nitro compounds to corresponding amines, where a synergistic role among Co, Zn, and N was responsible for its superior performance to other tested catalysts. In addition, the N-doped non-noble metal/carbon heterogeneous catalyst was fairly stable and could be reused several times without obvious deactivation.

## Introduction

With the progress of modern society, the increasing requirement of fine chemicals and other important materials such as dyes, pigments, food additives, pharmaceuticals, and herbicides have brought a series of problems, demanding prompt solution from scientists around the world (Li et al., 2018; Sudarsanam et al., 2019). Especially, aromatic or aliphatic amines are among the most significant fundamental molecules in these chemical industries (Zhou et al., 2017; Li et al., 2019). Generally, amines are produced by catalytic hydrogenation or transfer hydrogenation of nitro compounds in the presence of heterogeneous noble metal catalysts, which have drawbacks in price and reserve with complex preparation methods (Sudarsanam et al., 2018; Yuan et al., 2018). In addition, the utilization of gaseous H_2_ will give rise to a number of problems such as safety and transportation problems (Zeynizadeh et al., 2016; Formenti et al., 2019). Therefore, there are two urgent issues need to be solved: (1) seeking alternatives to gaseous hydrogen sources. (2) A simple and low-cost approach to prepare the catalyst featured with high efficiency and high stability for transfer hydrogenation of nitro compounds.

Formic acid (FA), derivable from biomass as well as CO_2_ reduction, is recyclable and sustainable, and has attracted increasing concerns in the hydrogenation of nitro compounds. The utilization of formic acid as hydrogen source seemed to be a good solution to solve the above-mentioned problems caused by H_2_ (Zhou and Zhang, 2017; Zhou et al., 2017; Du et al., 2018; Yuan et al., 2018), while it should be pointed out that the used catalyst must be stable enough to resist the acidic formic acid (Li et al., 2016). In this regard, it is highly desirable to design an efficient while robust catalyst to meet the above requirements.

Zeolitic imidazolate frameworks (ZIFs) are a branch of metal-organic frameworks (MOFs) materials in possession of carbon, nitrogen and transition metals within a highly porous structure (Salunkhe et al., 2016). The metal ions and imidazolate strongly coordinate with each other to keep the structural integrity of ZIFs, even being used in solvents. Moreover, the preparation process of this type of materials is very simple and inexpensive (Chen et al., 2015; Yang et al., 2015; Wang et al., 2016; Park et al., 2019). It was reported that the introduction of secondary metal nodes into the ZIF framework can further improve the catalyst performance in various fields (Yang et al., 2015; Yan et al., 2017; Bai et al., 2018; Wang et al., 2018), due to the formation of more defects in the catalyst framework or surface (Chen et al., 2018). Also, close contact between two metals can produce excellent synergistic effects and thus improve their inherent properties. However, it is rarely reported the use of bimetallic catalysts for the selective reduction of nitro compounds using formic acid as a hydrogen source.

Herein, a simple solvothermal method was utilized to prepare the Co-Zn-ZIF material, followed by high-temperature pyrolysis in N_2_ to get the mesoporous Co-Zn/N-C catalyst, which was proved to be highly efficient for hydrogenation of nitrobenzene to aniline using formic acid as a hydrogen donor. This developed solid catalyst was widely applicable to a wide range of nitro compounds while remained good stability and recyclability.

## Experimental Section

### Materials

All the used nitro and amino compounds, methanol (MeOH, 99%), ethanol (EtOH, 99%), tetrahydrofuran (THF, 99%), acetonitrile (CH_3_CN, 99%), and 1,2-dichloroethane (CH_2_ClCH_2_Cl, 99%), cobalt nitrate (98%), zinc nitrate (98%), copper nitrate (98%), nickel nitrate (98%), and 2-methylimidazole (98%) were purchased from Beijing Innochem Technology Co., Ltd. Acetonitrile (MeCN, 99%), dichloromethane (DCM, 99%), and xylene (99%) were purchased from Sigma-Aldrich Co. LLC. *N,N'*-Dimethylformamide (DMF, 99%) and dimethyl sulfoxide (DMSO, 99%) were purchased from Tianjin Kermel Co., Ltd.

### Preparation of Co-Zn/N-C-T

The ZIF analogous materials (Co-Zn/N-C-T, T denotes pyrolysis temperature of 600, 700, and 800°C) were prepared by using a solvothermal and pyrolysis method. With a general procedure, 0.5 mmol cobalt nitrate and 0.5 mmol zinc nitrate were dissolved into 40 mL ethanol in a 100 mL beaker, and the resulting mixture was labeled as solution A (magenta solution). Then, 8 mmol 2-methylimidazole with equivalent triethylamine was dissolved into 20 mL ethanol contained in a 150 mL round-bottom flask, marked as solution B (transparent solution). Solution A was dropwise added into solution B and stirred at 25°C for 0.5 h, and the purple precipitate was observed as A was added into B. Co-Zn-ZIF was obtained after centrifugation, washing with ethanol to neutral to ensure that triethylamine is moved away, then drying at 80°C overnight. Then, the resulting solid was subjected to pyrolysis at the specific temperature (800, 700, or 600°C) for 3 h with the heating ramp of 5°C/min in a nitrogen atmosphere. Upon cooling down to room temperature, the magnetic black powder Co-Zn/N-C-T was collected. For comparison, Co/N-C-T and Zn/N-C-T were also prepared via the same synthetic procedures by only changing the bimetal to single metal nitrate (1 mmol cobalt nitrate or zinc nitrate).

The employed nitrogen tube muffle furnace (OTF-1200X-50) was bought from Hefei Kejing materials Technology Co., Ltd. TG analysis manifests that the pyrolysis temperature of ≥ 600°C is enough for carbonization of Co-Zn-ZIF under pyrolysis conditions (Figure S1).

### Catalyst Characterization

The Micromeritics ASAP 2010 instrument (Tristar II 3020, Norcross, GA) was used to test BET surface areas of the as-prepared porous catalysts by nitrogen physisorption measurements at 77 K. An aberration-corrected FEI Tecnai G2 F20 S-TWIN (S) TEM (Hillsboro, OR) operating at 300 kV with the energy dispersive X-ray (EDX) spectra were used to get STEM-HAADF imaging. The Physical Electronics Quantum 2000 Scanning ESCA Microprobe (Physical Electronics Inc., PHI, MN) equipped with a monochromatic Al Kα anode was used to measure the XPS (X-ray photoelectron spectroscopy) of the as-prepared catalysts. D/max-TTR III X-ray powder diffractometer (Rigaku International Corp., Tokyo) using Cu Kα radiation source was utilized to test the XRD (X-ray diffraction) patterns of different catalysts. The Renishaw RM2000 was employed to collect Raman spectra at room temperature from 100 to 3,000 cm^−1^ with 532 nm argon ion laser. Inductively coupled plasma optical emission spectroscopy (ICP-OES) analysis was conducted using a PerkinElmer Optima 8000 instrument. The thermal gravimetric (TG) measurements were conducted by Shimadzu DTG-60AH differential thermal analyzer.

### Transfer Hydrogenation of Nitro Compounds

All the catalytic transfer hydrogenation reactions were carried out in 15 mL Ace pressure tube. Firstly, 0.2 mmol nitro compound, 20 mg Co-Zn/N-C (5 mol% Co), 2.0 mL THF, and 6 equiv. HCOOH were added into the tube and sealed. Then, the tube was transferred into an oil bath preheated to the desired reaction temperature and magnetically stirred at 600 rpm for specific reaction time. The time was recorded as soon as the tube was placed into the oil bath. When the reaction time was up to the required time, the tube was taken out and cooled down to ambient temperature using flow tap-water. Then, 3 mL THF and 10 mg naphthalene as internal standard were added into the reaction mixture.

### Product Analysis

Upon the completion of the reaction, the filter membrane was used to remove solid particles from the liquid solution, followed by quantitative analysis with gas chromatography (GC Agilent 7890B with an HP-5 column (30 m × 0.320 mm × 0.25 μm) and FID). The quantification of the obtained products was conducted by referring to the standard curves (*R*^2^ > 0.99) made from commercial samples. The product structures were identified by gas chromatography-mass spectrometry (GC-MS Agilent 6890N GC/5973 MS). Then, nitrobenzene conversion (*C*, %) and aniline yield (*Y*, %) for the model reaction were calculated according to the below equations:

$$\begin{array}{r} C\;=\left[1-\frac{mole\;concentration\;of\;nitrobenzene\;in\;product}{mole\;concentration\;of\;initial\;nitrobenzene}\right]\\ \;\times 100\%\\ \;Y\;=\left[\frac{mole\;concentration\;of\;aniline}{mole\;concentration\;of\;initial\;nitrobenzene}\right]\\ \;\times 100\% \end{array}$$

## Results and Discussion

### The Characterization Results

**Figure 1:**
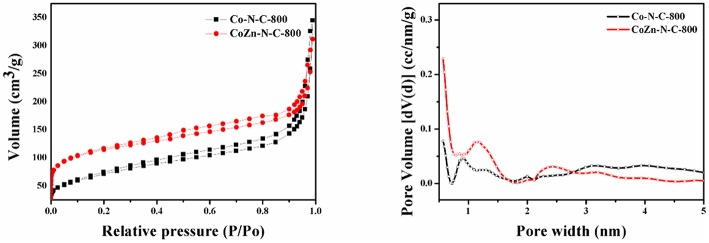
Nitrogen adsorption-desorption isotherms and corresponding pore size distributions calculated by the DFT model of the Co /N-C-800 and Co-Zn/N-C-800 at 77 K.

The nitrogen adsorption-desorption isotherms and pore distribution of Co-N-C-800 and CoZn-N-C-800 are shown in Figure 1. It can be clearly found that all the catalysts exhibit mesoporous structures in consideration of type-IV curve and H4-type hysteresis loop at the relative pressure P/P_0_ of 0.4–1.0. Among them, the Co-Zn/N-C-800 sample reveals relatively large volume (average pore diameter 12.7 nm), and the pore distribution also shows that the pore width of Co-Zn/N-C-800 is mainly located in the range of 0–1.5 nm and 2–50 nm (Figure S2). Meanwhile, the BET surface area of Co-Zn/N-C-800 is 411.3 m^2^/g, which is larger than that of Co/N-C-800 (257.1 m^2^/g). It is expected that cobalt can be reduced while zinc is partially evaporated to generate additional holes in the pyrolysis process at 800°C and N_2_ flow, thus increasing the surface area.

**Figure 2:**
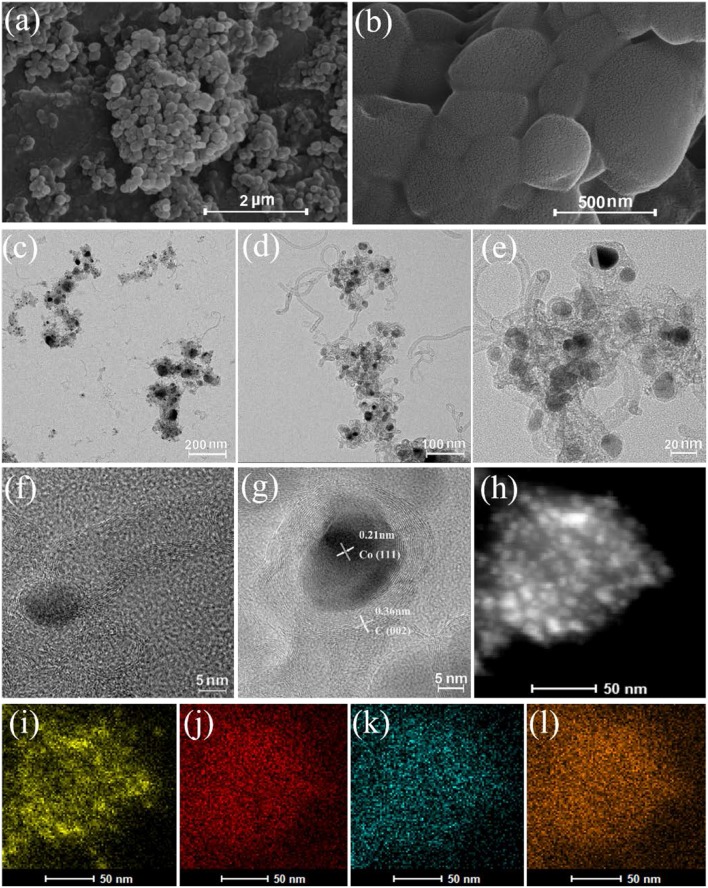
SEM images of Co-Zn/N-C-800 **(a,b)**, TEM images of for Co/N-C-800 **(c)**, and Co-Zn/N-C-800 **(d,e)**. HRTEM images of Co particle and C graphic of Co-Zn/-N-C-800 **(f,g)**, STEM-HAADF image **(h)**, and Co: yellow **(i)**, C: red **(j)**, Zn: blue **(k)**, and N: orange **(l)** mappings of Co-Zn/N-C-800.

To more intuitively understand the catalyst morphology, SEM, TEM, and STEM-HAADF were utilized to characterize Co/N-C-800 and Co-Zn/N-C-800 (Figures 2a–f). Both materials have the tubular graphene structure and the metal particles are fully dispersed on the carbon carrier, which is consistent with a previous report. The metal nanocatalysts would be generated from the reduction of the metal ions/clusters with reducing gases (e.g., CO) *in situ* generated from the pyrolysis of organic species, and these metal nanocatalysts were capable of further catalyzing the organic units to form the graphene structures and *N*-doped carbon nanotubes (Chen et al., 2008; Xia et al., 2016; Gong et al., 2019). The formed graphene structure can effectively enhance the resistance of the catalyst to acid, thus possibly improving its catalytic activity. The lattice fringe images of the catalyst show the formation of graphitic carbon C (002) plane with 0.36 nm and Co (111) plane with 0.21 nm (Figure 2g). Also, the elemental mapping of Co-Zn/N-C-800 clearly states the well-proportioned distribution of Co, N, and C at the nanoscale (Figures 2h-l) (Co: yellow, C: red, Zn: blue, N: orange).

**Figure 3:**
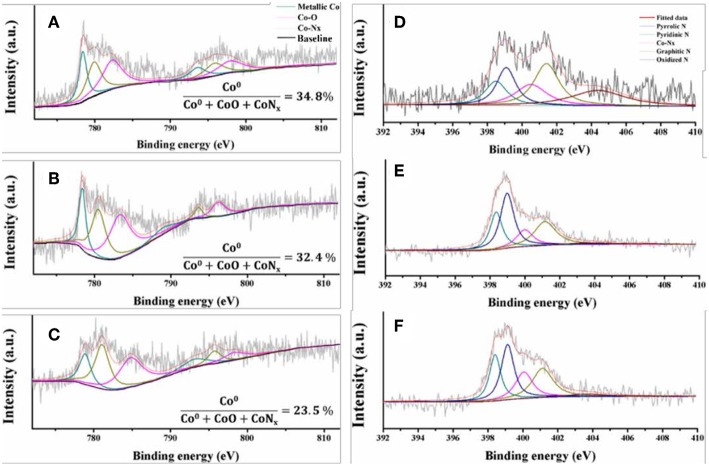
High resolution XPS spectra of Co 2p of Co/N-C-800 **(A)** fresh Co-Zn/N-C-800 **(B)**, reused Co-Zn/N-C-800 (after the fifth recycle) **(C)** and N 1s of Co/N-C-800 **(D)** fresh Co-Zn/N-C-800 **(E)**, reused Co-Zn/N-C-800 **(F)**.

**Figure 4:**
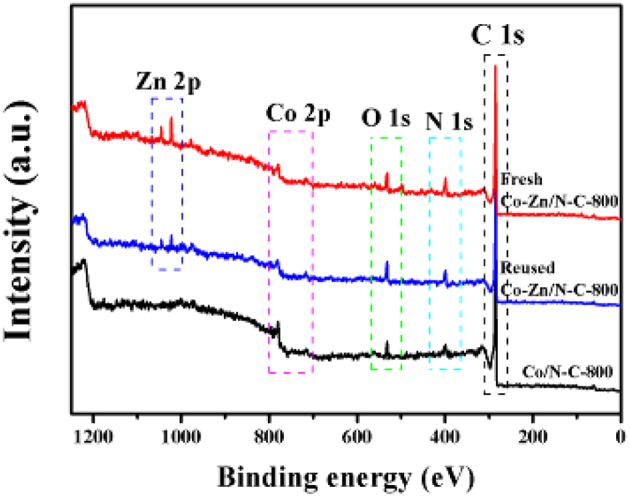
Wide-range XPS spectra of fresh Co-Zn/N-C-800, reused Co-Zn/N-C-800 (after the fifth recycle) and Co/N-C-800.

The Co/N-C-800 and Co-Zn/N-C-800 catalysts were further characterized by XPS, and the obtained high-resolution XPS spectra of Co_2p_ of Co/N-C-800, fresh Co-Zn/N-C-800, and reused Co-Zn/N-C-800 are shown in Figure 3. Metallic Co (green line), Co-O (magenta line) and Co-N_*x*_ (yellow line) were detected, and located at 778.4, 781.7, and 780.0 eV, respectively (Park et al., 2019). Compare to that of Co/N-C-800, a negative Co_2p_ peak shift in the fresh and reused Co-Zn/N-C-800 catalysts could be observed, possibly due to the incorporation of Zn species (Wu et al., 2018). Based on the integral area of Co in different valence states, the percentage composition of the metal Co content is also listed in this figure. No significant difference in the metallic Co content between Co/N-C-800 (34.8%) and Co-Zn/N-C-800 (32.4%). These results demonstrate the tight interaction between the metal species Co and the nitrogen species. XPS spectra of N_1s_ illustrates three kind of nitrogen species, including pyridine N 398.5 eV (deep green line) pyrrolic N 400.1 eV and graphic N 401.1 eV (yellow line) that probably combined with metallic Co and fixed in the catalyst structure (Figures 3D–F), which is well supported by the XPS spectra of Co_2p_ (Figures 3A–C). The presence of graphic N can find a clue from the formed graphene structure, as verified by TEM (Figure 2). Also, the XPS spectra of C_1s_ (shown in Figure S3) show three peaks at 284.6, 285.8, and 288.6 eV, illustrating the existence of C = C/C-C, C-N, and N = C-N bonds in the materials, respectively (Shen et al., 2017; Zhao et al., 2017). In addition, the existence of the Zn element in fresh Co-Zn/N-C-800 and even the one reused for five times was affirmed by the XPS spectra of Zn_2p_ peak (Figure 4), which is consistent with ICP analysis (ca. 20% Zn left after pyrolysis).

**Figure 5:**
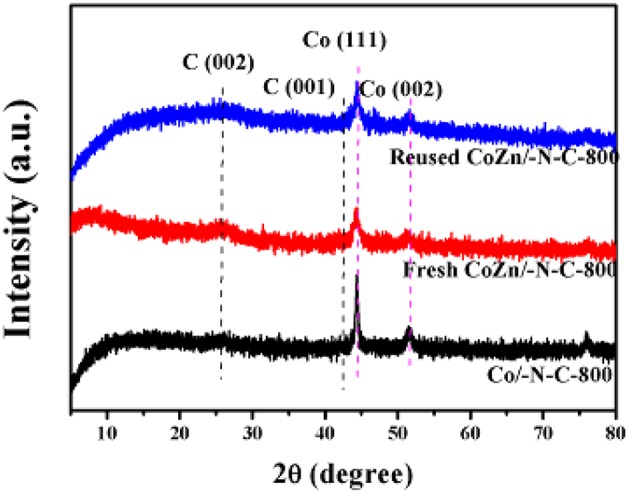
The XRD patterns of reused Co-Zn/N-C-800 (after the fifth recycle), fresh Co-Zn/N-C-800, and Co/-N-C-800.

XRD patterns (Figure 5) show a significant peak at 25.3 degree (C 002) in those three materials (fresh Co-Zn/N-C-800, reused Co-Zn/N-C-800 and Co/N-C-800), which is assigned to the graphitized carbon, while the peaks at 44.3° and 51.4° belong to Co (111) and Co (002) respectively, which is strongly agreeing with the characterization results of TEM and XPS.

**Figure 6:**
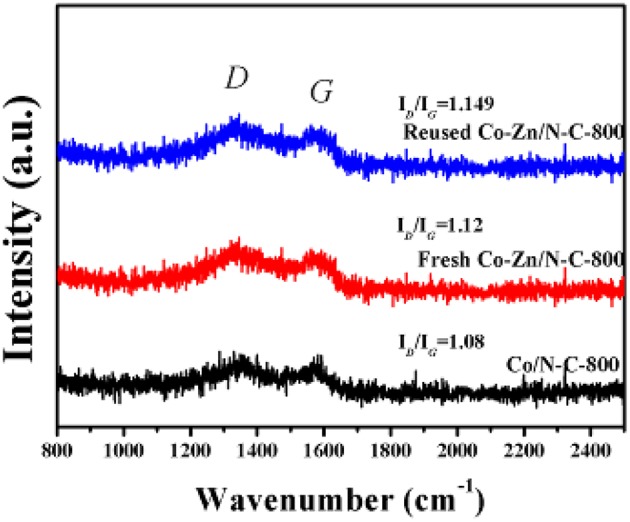
The Raman spectra of reused Co-Zn/N-C-800 (after the fifth recycle), fresh Co-Zn/N-C-800, and Co/-N-C-800.

Raman spectra of those three samples are provided in Figure 6, and the D and G peaks are located at 1,345 cm^−1^ and 1,570cm^−1^, respectively. The relative peak intensity of D and G (I_*D*_/I_*G*_) represents the degree of the graphitization of carbon materials (Wang et al., 2017). It can be seen that the I_D_/I_G_ ratios of reused Co-Zn/N-C-800, fresh Co-Zn/N-C-800, and Co/N-C-800 are 1.149, 1.12 and 1.08, respectively, suggesting that Co/N-C-800 without the inclusion of Zn has a higher degree of graphitization than both fresh and reused Co-Zn/N-C-800 catalysts. That is to say, the fresh Co-Zn/N-C-800 has more defects compared to the Co/N-C-800. In addition, the reused Co-Zn/N-C-800 has a slightly increased value of I_*D*_/I_*G*_ ratios, as compared with the fresh counterpart. It is indicated that the structural defects of the catalyst increase after recycle.

### Catalytic Testing Results

The influence of different catalysts on transfer hydrogenation of nitrobenzene is shown in Table 1. It was found that Co/N-C-800 gave 71.0% nitrobenzene conversion and 57.6% aniline yield. When Co-Zn/N-C-800 was used instead of Co/N-C-800, relatively high nitrobenzene conversion of 83.9% and 75.6% aniline yield could be achieved. This result shows that the introduction of Zn into the catalyst may enhance its productivity and selectivity. Meanwhile, the residue of Zn might contribute to the enhanced productivity, due to the synergistic effect of Zn and Co species in the hydrogen transfer process (Kong et al., 2017). In contrast, 3.2% nitrobenzene conversion and <1% aniline were obtained with Zn/N-C-800, indicating that the presence of Zn does have a promotional effect on the reaction.

**Table 1:** Transfer hydrogenation of nitrobenzene with different catalysts^a^.

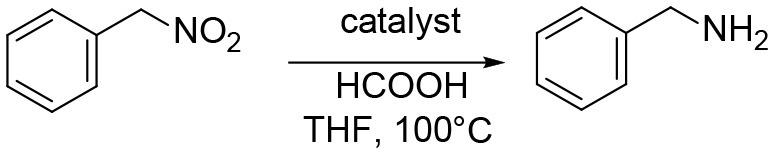			
**Catalyst**	**Pyrolysis condition (N**_**2**_**)**	**Conv. (%)**	**Yield (%)**
Co/N-C	800°C, 3 h	71.0	57.6
Co/N-C	700°C, 3 h	62.3	47.9
Co/N-C	600°C, 3 h	38.8	11.6
Co-Zn/N-C	800°C, 3 h	83.9	75.6
Co-Zn/N-C	700°C, 3 h	43.8	34.0
Co-Zn/N-C	600°C, 3 h	58.8	<1
Co-Zn/N-C	900°C, 3 h	76.6	65.6
Zn/N-C	800°C, 3 h	3.2	<1
Zn/N-C	700°C, 3 h	5.1	<1
Zn/N-C	600°C, 3 h	7.9	<1
N-C	800°C, 3 h	<1	<1
N-C	700°C, 3 h	<1	<1
N-C	600°C, 3 h	<1	<1

*^a^Reaction conditions: 0.2 mmol nitrobenzene, catalyst (5 mol% Co), 1.2 mmol HCOOH, 2 mL THF, 2 h, 100°C, 600 rpm*.

On the other hand, the pyrolysis temperature also plays a vital role in the catalyst reactivity (Table 1). With the decrease of the pyrolysis temperature for Zn-N-C from 800°C to 700 or 600°C, the conversion of nitrobenzene decreases gradually (from 7.9 to 3.2%). It can be deduced that the rise of temperature may be favorable for the evaporation of Zn from the catalyst. In this regard, the pre-introduction of Zn into the Co catalyst may increase the catalyst surface area and pore size. This conclusion in agreement with the result of BET analysis of Co-Zn/N-C-800 and Co/N-C-800. However, for the system with Co/N-C as catalysts, the yield of aniline and conversion of nitrobenzene increase (from 11.6 to 57% and <1 to 75.6%) with the rise of pyrolysis temperature from 600 to 800°C. The enhanced reaction performance may be correlated with more Co^2+^ to Co^0^ being reduced at relatively high pyrolysis temperatures (Wang et al., 2014). It should be mentioned that 76.6% nitrobenzene conversion and 65.6% aniline yield were achieved over Co-Zn/N-C-900, which is inferior to those of Co-Zn/N-C-800 (Table 1), indicating that a relatively higher pyrolysis temperature (> 800°C) has a negative effect on catalytic performance. These results imply that a relatively high pyrolysis temperature (e.g., 800°C) is essential to achieve satisfactory result s, wherein the partial removal/evaporation of Zn from the precursor at that temperature may improve the texture and electronic structures (Dai et al., 2017).

**Figure 7:**
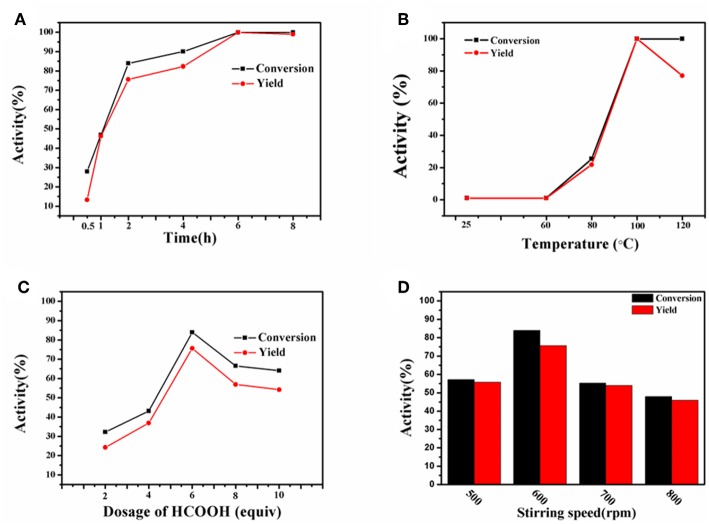
Transfer hydrogenation of nitrobenzene with varying time **(A)**, temperature **(B)**, amount of HCOOH **(C)** and stirring speed **(D)**. Reaction conditions: 0.2 mmol nitrobenzene, Co-Zn/N-C-800 (5 mol% Co).

In order to get optimal reaction conditions for transfer hydrogenation of nitro compound, the effect of temperature, time, and the dosage of HCOOH on the conversion of nitrobenzene to aniline reaction was initially investigated. Figures 7A,B collects the results obtained at the reaction temperature of 25 to 120°C within the variable reaction time of 0.5 to 8 h. It was not difficult to see that both reaction parameters have a great influence on the catalyst activity. As the reaction was carried out at 100°C for 0.5 h, only 13.3% aniline yield and 32.3% nitrobenzene conversion could be obtained. With gradually prolonging the reaction time, a maximum aniline yield of 99.9% at 99.9% nitrobenzene conversion could be attained at 100°C after 6 h, while continuing to increase the reaction duration to 8 h, the yield slightly decreases to 99.0%, due to the formation of formamide from aniline. Therefore, the optimum reaction time for the synthesis of aniline is 6 h. However, inferior yield and conversion were achieved at a relatively lower temperature of 25 and 60°C in 6 h. Further increasing the reaction temperature to 100 and 120°C, the yield of aniline first increases to 99.9% but then decreases to 77.0 % with the almost constant conversion of 99.9%, in which the decline in aniline yield is ascribed to the conversion of aniline to formamide. As a result, 100°C was screened out as the optimum reaction temperature. In addition, the effect of HCOOH amount (2 to 10 equiv. relative to the substrate) on the production of aniline was investigated at 100°C for 2 h (Figure 7C). With the increase of HCOOH amount from 2 to 6 equiv., both the yield and conversion gradually increase to 75.6 and 83.9%, but further increasing the dosage of HCOOH cause the decline in the catalytic activity. The more HCOOH is used, the side reaction with aniline is more prone to take place. Thus, 6 equiv. HCOOH was the optimized amount suitable for the reaction.

**Figure 8:**
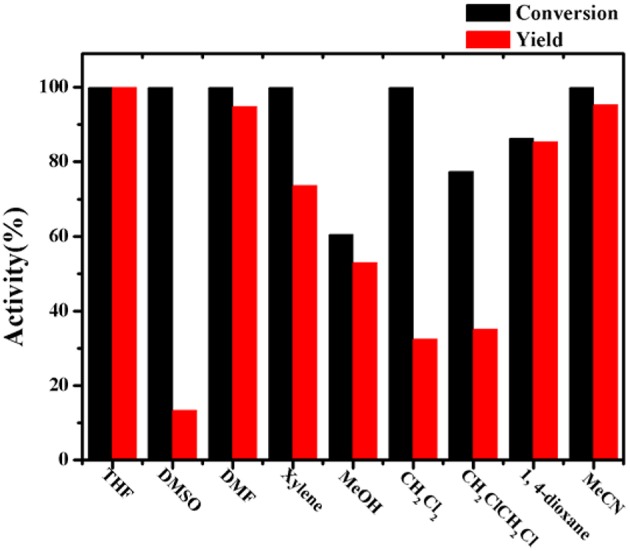
Transfer hydrogenation of nitrobenzene with different solvents. Reaction condition: 0.2 mmol nitrobenzene, Co-Zn/N-C-800 (5 mol% Co), 2 mL solvent, 1.2 mmol HCOOH, 100°C, 6 h, 600 rpm.

Since the obtained Co-Zn/N-C is magnetic after pyrolysis, it prefers to adhere to the magneton during stirring. In this regard, it is necessary to select a suitable stirring rate (500 to 800 rpm) so that the catalyst can sufficiently disperse in the reaction system, thus maximizing the reaction activity. As shown in Figure 7D, 57.2 % conversion and 55.7 % yield were obtained at 500 rpm. When the stirring rate was increased to 600 rpm, a high nitrobenzene conversion (83.9%) and aniline yield (75.6%) could be achieved. Unexpectedly, a dramatic decline in activity was observed when the stirring rate continued increasing to 700 and 800 rpm, where a high rotation speed causes the magnetic catalyst being thrown to the bottle wall of the Ace pressure tube, thus making the catalyst be isolated from the reaction system. So it can be concluded that a low stirring rate cannot fully disperse the magnetic catalyst into the reaction system, while an excessive stirring rate causes the catalyst to be separated out from the reaction system, both of which will reduce the activity. Overall, the 600 rpm seems to be the best stirring rate in this system, and the images of each reaction system at different stirring speeds were exhibited in Figures S4, S5. For solvents used in the reaction system, the catalytic results obtained with THF, DMSO, DMF, xylene, MeOH, CH_2_Cl_2_, 1, 4-dioxane, and MeCN are shown in Figure 8, and THF is found to be favorable for aniline production with 99.9% yield.

**Figure 9:**
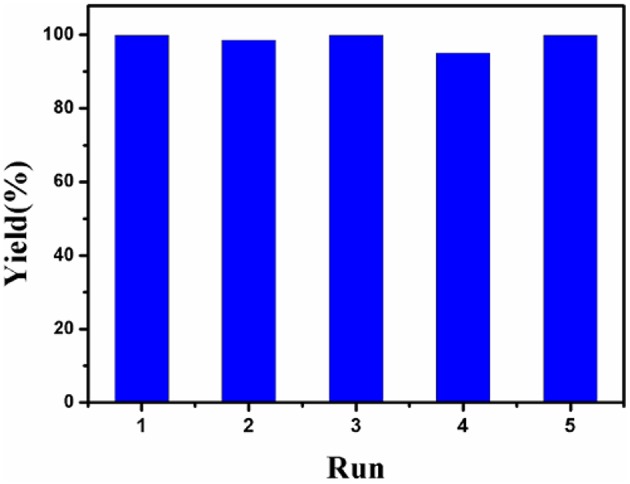
The recyclability tests of Co-Zn/N-C-800. Reaction conditions: 0.2 mmol nitrobenzene, Co-Zn/N-C-800 (5 mol% Co), 1.2 mmol HCOOH, 2 mL THF, 100°C, 6 h, 600 rpm.

The recyclability of Co-Zn/N-C-800 catalyst under optimized reaction conditions (i.e., 100°C, 6 h,) was tested and provided in Figure 9. After each cycle of reaction, the Co-Zn/N-C-800 catalyst was collected and separated by an external magnet (Figure S3), followed by washing with THF and ethanol for three times (3 × 5 mL), and drying at 80°C overnight, which was then directly used for the next run. The catalyst activity has no obvious decrease after five consecutive reaction runs, while suddenly declines (75.6% yield) in the sixth cycle, subsequently. The catalyst deactivation results from the deposition of organic species during the reaction. XPS analysis discloses that the cobalt content in the Co-Zn/N-C-800 catalyst decreased from 32.4 to 23.5% after reusing for fifth times (Figures 3A,C), indicating that the occurrence of adsorbing organic species during the reaction and the leaching of Co species may contribute to the slight deactivation of the catalyst during the recycles. The XRD patterns show that the crystalline phase of reused Co-Zn/N-C-800 does not change obviously. In addition, TEM illustrates the negligible aggregation of Co-Zn/N-C-800 after reusing for five cycles (Figure S6), and mostly maintained its original structure. These catalyst characterization data indicated that the Co-Zn/N-C-800 material is stable in the acidic reaction system and could keep an excellent catalytic activity for the hydrogenation of nitro-compounds even after five reaction cycles.

Finally, to examine the practicability and generality of Co-Zn/N-C-800 in the transfer hydrogenation reaction, a variety of nitro compounds were employed as substrates and the obtained results are shown in Table 2. Gratifyingly, good to excellent yields of corresponding aromatic amines could be obtained under the relatively mild reaction conditions.

**Table 2:** Hydrogenation of different nitro compounds with Co-Zn/N-C-800 catalyst^a^.

**Entry**	**Substrate**	**Product**	**Time (h)**	**Conv. (%)**	**Yield (%)**
1	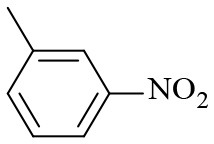	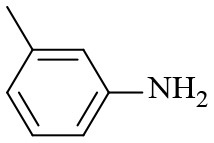	6	> 99.9	> 99.9
2	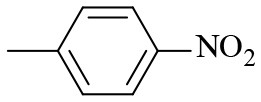	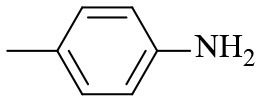	6	94.3	92.9
3	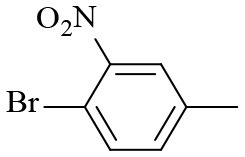	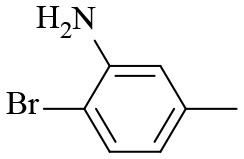	6	83.5	81.2
4	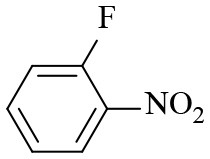	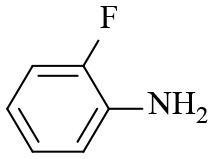	6	> 99.9	> 99.9
5	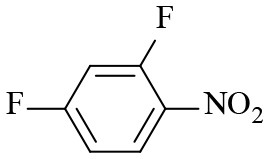	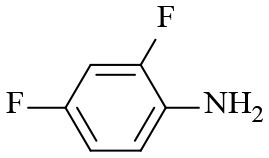	6	> 99.9	> 99.9
6	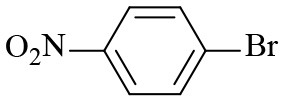	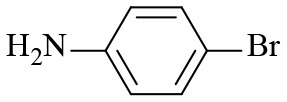	6	> 99.9	> 99.9
7	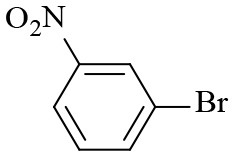	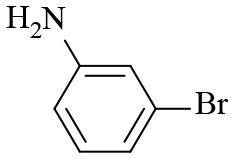	6	> 99.9	> 99.9
8	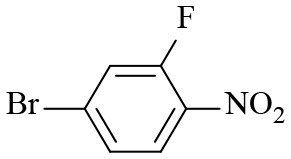	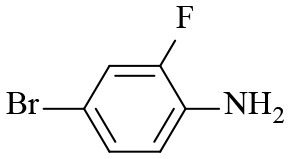	6	97.2	90.1
9	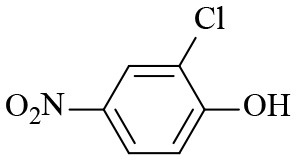	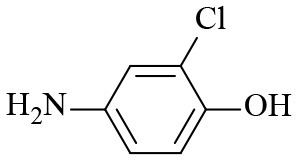	10	55.4	53.0
10	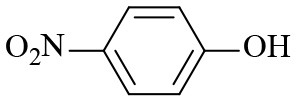	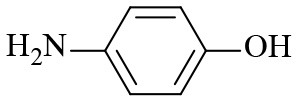	10	98.3	97.0

*^a^Reaction conditions: 0.2 mmol substrate, Co-Zn/N-C-800 (5 mol% Co), 2 mL THF, 1.2 mmol HCOOH, 100°C*.

## Conclusions

A new type of efficient and segregative Co-Zn/N-C-800 catalysts was prepared by a facile solvothermal and subsequent high-temperature pyrolysis method. Under optimum reaction conditions (100°C, 6 h), 99.9% nitrobenzene conversion and 99.9% aniline yield could be obtained. In addition, Co-Zn/N-C-800 also showed pronounced catalytic reactivity in the transfer hydrogenation of various nitro compounds, wherein a synergistic role among Co, Zn, and N was found to be responsible for its superior performance and the forming graphene structure can effectively protect the active site from the corrosion of formic acid. In addition, this N-doped non-noble metal/carbon heterogeneous catalyst was comparatively stable and could be reused for several times without obvious deactivation. These findings of our research provide an ideal reference for rational manufacture of the bimetallic catalyst that can remarkably enhance the catalyst reactivity while maintaining the catalyst stability.

## Data Availability

The raw data supporting the conclusions of this manuscript will be made available by the authors, without undue reservation, to any qualified researcher.

## Author Contributions

YX performed experiments and wrote the manuscript. HL and SY were in charge of designing the experiments and revising the manuscript. JL and WZ assisted YX in completing part of experiments.

### Conflict of Interest Statement

The authors declare that the research was conducted in the absence of any commercial or financial relationships that could be construed as a potential conflict of interest.
