# Supplementary material for: Efficient Transfer Hydrogenation of Nitro Compounds to Amines Enabled by Mesoporous N-Stabilized Co-Zn/C
Source: Front Chem. 2019 Aug 27;7:590. doi: 10.3389/fchem.2019.00590 (PMC6718455; doi:10.3389/fchem.2019.00590)

**Supplemental information**

**Efficient transfer hydrogenation of nitro compounds to amines enabled by mesoporous N-stabilized Co-Zn/C**

**

**

**Fig. S1** TG curve of fresh Co-Zn/N-C-800


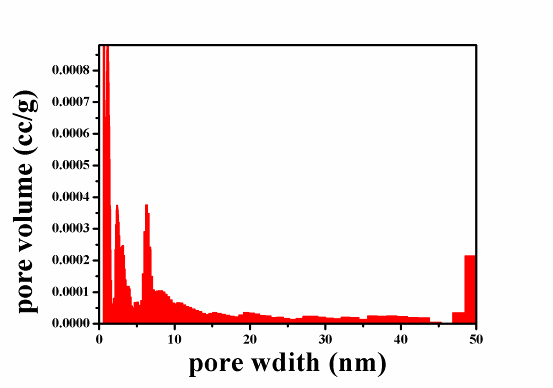

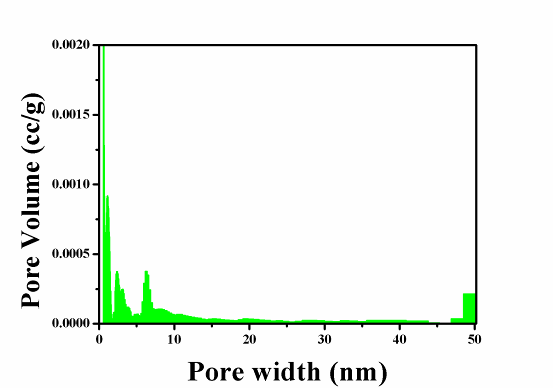


**Fig. S2** Pore distribution of Co-Zn/N-C-800 (red) and Co/N-C-800 (green)





**Fig. S3** The high resolution XPS of C 1s of reused Co-Zn/N-C-800, Co/N-C-800, and fresh Co-Zn/N-C-800.


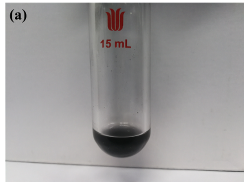

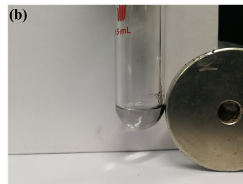


**Fig. S4** The image of magnetism presentation of Co-Zn/N-C-800


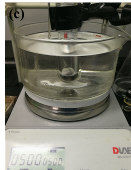

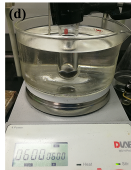

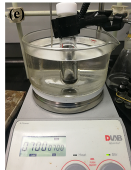

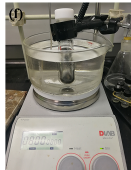


**Fig. S5** The Images of reaction systems with different stirring speeds: 500 rpm (c), 600 rpm (d), 700 rpm (e), 800 rpm (f).


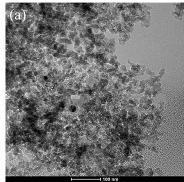

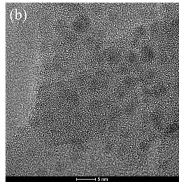


**Fig S6.** The high resolution TEM images of reused Co-Zn/N-C-800.

**GC-MS spectra of different products**


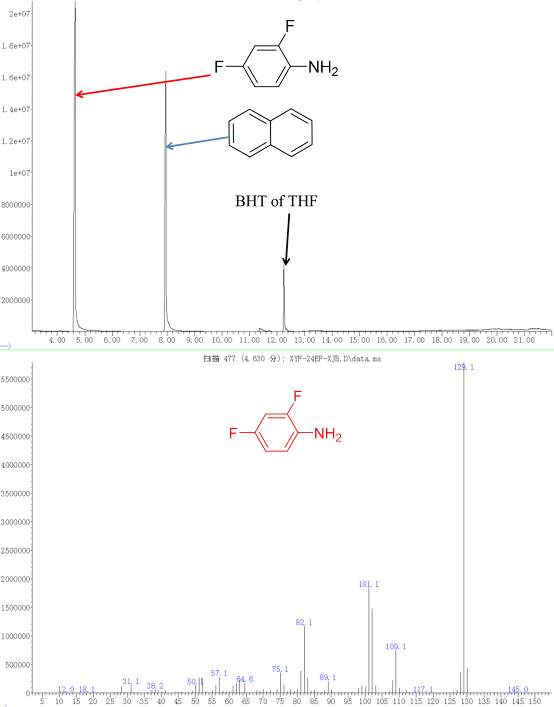


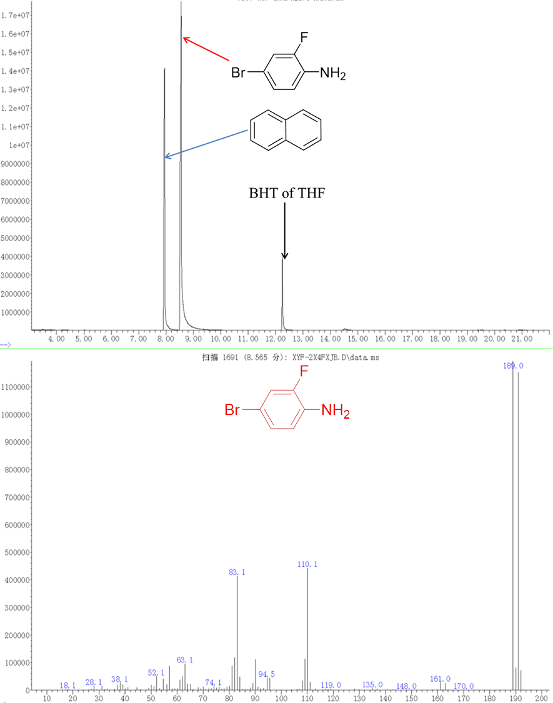

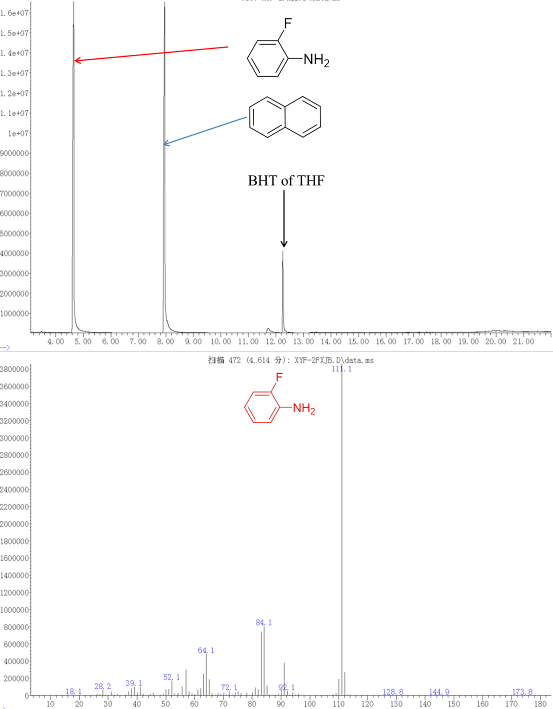

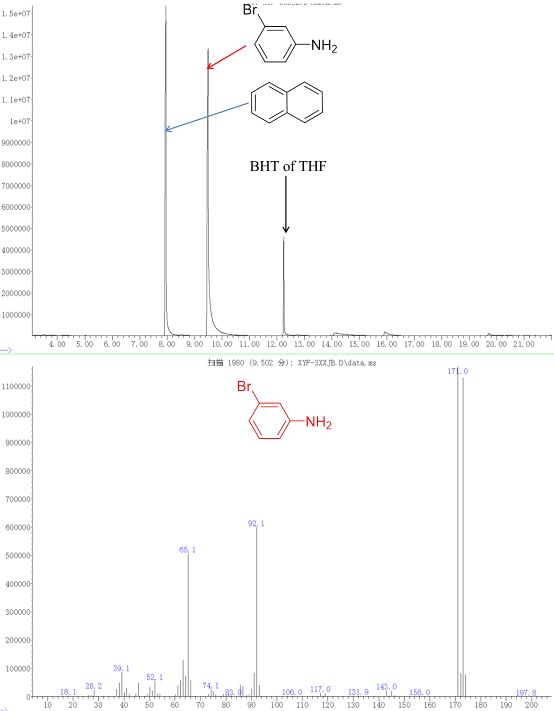

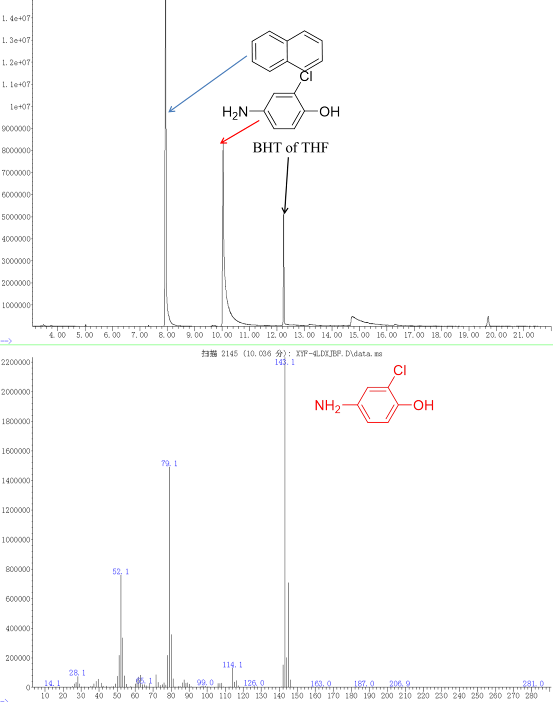

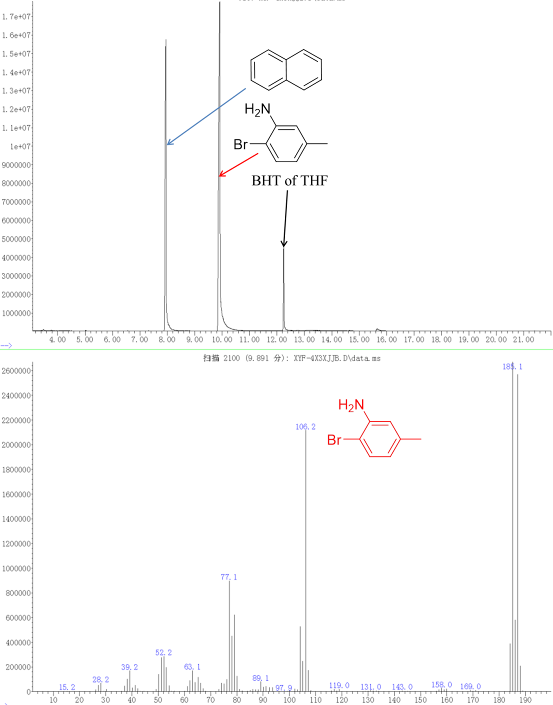

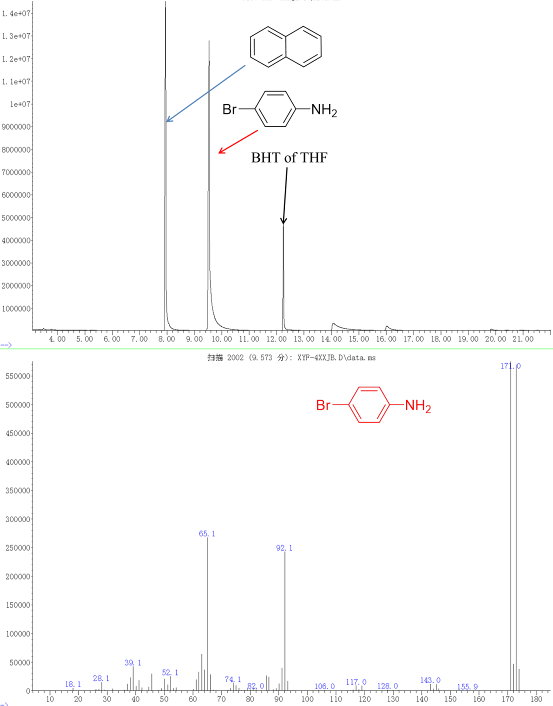

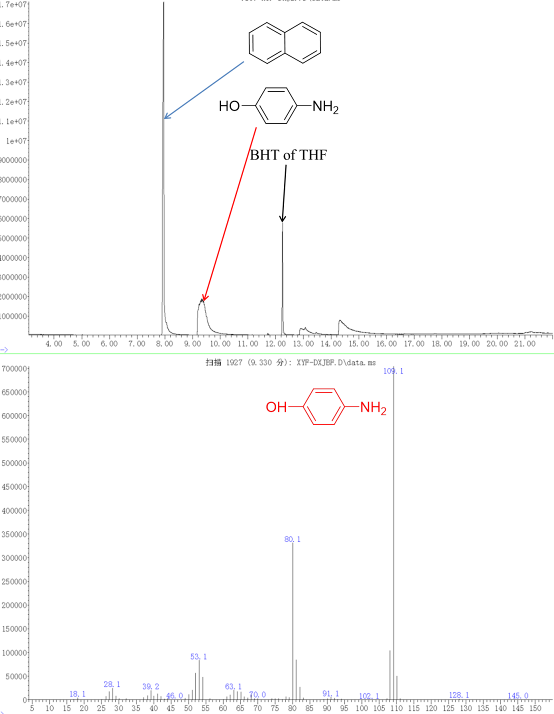

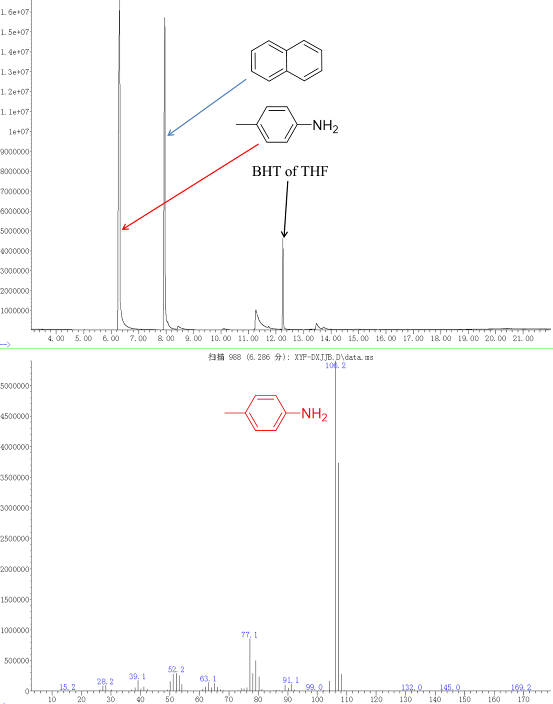

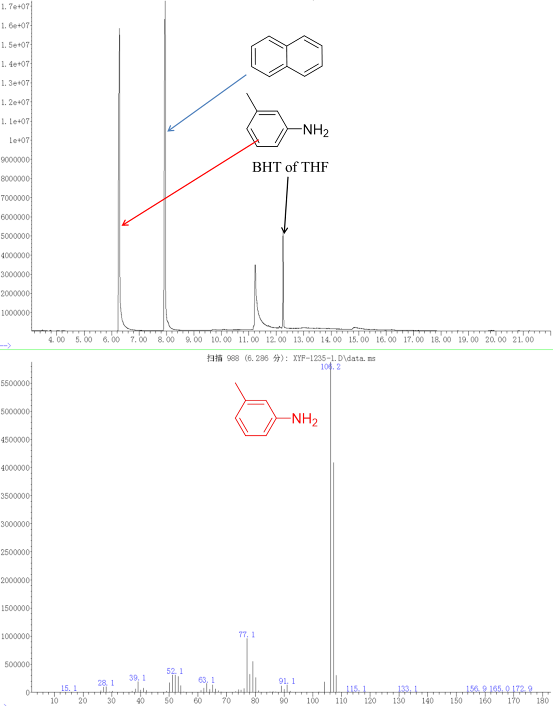

Supplement: Supplementary file 1 [file Data_Sheet_1.docx]
